# Profunda Brachii Pseudoaneurysm following Supracondylar Fracture of Humerus Repair in an 8-Year-Old Boy: A Case Report and Review of Literature

**DOI:** 10.1155/2021/1768529

**Published:** 2021-10-31

**Authors:** Hamed Ghoddusi Johari, Mohammad-Ali Erfani, Amirhossein Erfani, Reza Shahriarirad, Mohammad-Mehdi Karami

**Affiliations:** ^1^Thoracic and Vascular Surgery Research Center, Shiraz University of Medical Sciences, Shiraz, Iran; ^2^Trauma Research Center, General Surgery Department, Shiraz University of Medical Sciences, Shiraz, Iran; ^3^Research Center for Bone and Joint Diseases, Department of Orthopedic Surgery, Chamran Hospital, Shiraz University of Medical Sciences, Shiraz, Iran; ^4^Student Research Committee, Shiraz University of Medical Sciences, Shiraz, Iran

## Abstract

Arterial pseudoaneurysm can manifest in almost all arteries, but peripheral ones such as brachial artery pseudoaneurysm are rare and typically happen after trauma or infections. We describe an 8-year-old boy who presented with a large nonpulsatile soft tissue mass-like lesion 20 days after supracondylar fracture of the humerus which was fixed using Kirschner wire. The neurovascular examination was normal; CT angiography revealed a large pseudoaneurysm arising from the left profunda brachii artery. The patient went under surgical repair and was discharged from the hospital with an uneventful postop course. A high index of suspicion is necessary in cases with humeral fractures for the early diagnosis of pseudoaneurysm where the delayed diagnosis may cause fatal outcomes.

## 1. Introduction

Arterial pseudoaneurysm can manifest in almost all arteries, but peripheral ones such as brachial artery aneurysms are rare and typically happen after trauma or infections [1, 2]. Supracondylar fracture of the humerus accounts for almost 17.9% of fractures in children aged under 16 and can lead to vascular injuries in 10-14% of cases [3, 4]. Brachial artery pseudoaneurysm can often present as a mass-like lesion with rapid expansion and pain or ischemic complications due to thrombosis or distal embolization [1]. There have been several reports of iatrogenic pseudoaneurysm of the brachial artery. However, to the best of our knowledge, this is the first report of profunda brachii pseudoaneurysm followed by pediatric supracondylar fracture of the humerus. Here, we present a case pseudoaneurysm of profunda Brachii caused by injury after treatment of the supracondylar fracture of the humerus using Kirschner wire fixation.

## 2. Case Presentation

**Figure 1:**
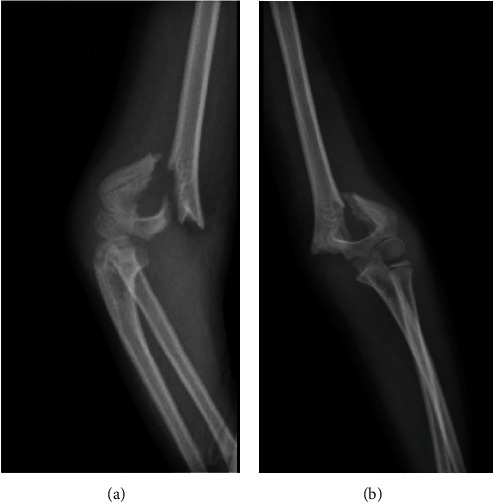
Supracondylar fracture of humerus with posterior displacement on first admission. Lateral view (a) and anterior posterior view (b).

**Figure 2:**
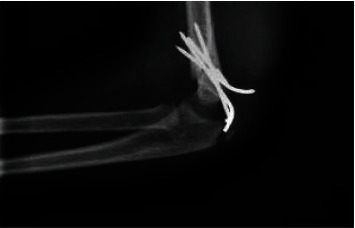
Lateral view of supracondylar fracture of humerus fixed with Kirschner wire (post-op).

**Figure 3:**
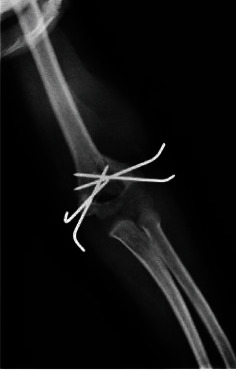
Anterior posterior view of supracondylar fracture of humerus fixed with Kirschner wire (post-op).

**Figure 4:**
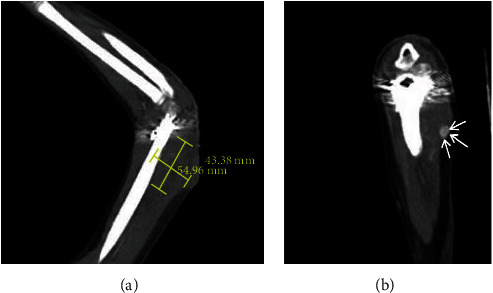
(a) Coronal cut of CT angiography demonstrating maximum diameter of sac of pseudoaneurysm originating from left profunda brachii artery. (b) Extravasation of contrast in pseudoaneurysm of profunda brachii artery in CT angiography.

**Figure 5:**
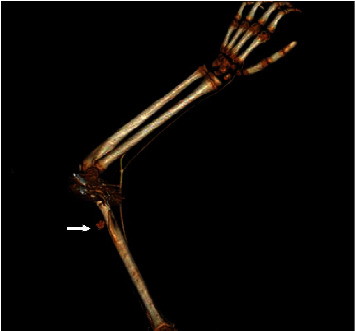
3D reconstruction of CT angiography demonstrating a pseudoaneurysm of profunda brachii artery with extravasation of contrast (white arrow).

An 8-year-old boy with no significant past medical history sustained a closed supracondylar fracture of the left humerus due to falling down. The initial X-ray demonstrated a type III fracture based on Gartland classification (Figure 1). He was admitted to the orthopedic ward in order to continue the treatment. Since closed reduction failed, the patient underwent surgery through posterior approach, patient being positioned in a lateral decubitus position and with the elbow held hyperflexed, in posterior aspect of elbow, a straight midline posterior incision was done, and medial and lateral skin flaps were created, elevated, and retracted. In medial aspect, ulnar nerve was isolated and protected, both medial and lateral paratricipital approach was done, and fracture site was exposed. Then, the fracture was reduced and with the first try, and 4 crossed Kirschner wires were inserted under direct visualization to achieve desired stability and rigidity. The patient was discharged the day after surgery (Figures 2 and 3). After 20 days, the patient was revisited due to pain and a large soft tissue mass-like lesion on the lateral aspect of the left elbow in site of surgery with no fever or other symptoms which was progressed gradually after being discharged. Physical examination revealed tense swollen sites of surgery with wound dehiscence and marginal necrosis with no evidence of infection or discharges. The patient was afebrile with a blood pressure of 110/70 mmHg, heart rate of 79, and a hemoglobin level of 8.3 g/dl. The neurovascular examination was normal with tenderness on a mass-like lesion without bruit. Based on preliminary assumption of hematoma formation versus pseudoaneurysm, soft tissue and duplex sonography were performed, which revealed normal arterial flow in all major arteries from the subclavian artery, brachial, and radial as well as ulnar arteries with a large collection in the posterior aspect of the elbow. However, based on the patient's history of recent trauma and physical examination, we were highly suspicious of pseudoaneurysm. Therefore, CT angiography was done, which revealed a huge pseudoaneurysm originating from the left profunda brachii artery with extravasation of contrast with the largest diameter of 54.96 mm (Figures 4 and 5).

**Figure 6:**
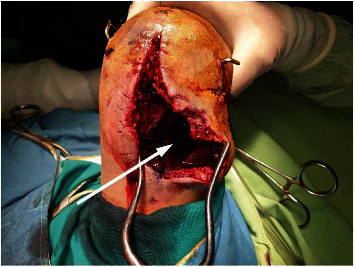
Open wound demonstrating the pseudoaneurysm sac through posterolateral approach. Neck of the pseudoaneurysm is visible in the depth of the wound (pointed by the arrow).

Based on the necrotic overlying skin, the patient was emergently transferred to the operation room in which after heparinization and proximal control by a tourniquet, and the sac of pseudoaneurysm was opened and approached through a left lateral arm incision (Figure 6). The neck of pseudoaneurysm originated from the profunda brachial artery 2 cm above the line of fracture which was in close contact with the tip of Kirschner wire making an exit from the medical cortex of the bony structure. Furthermore, the punctured site of profunda brachial artery was repaired by prolene oversewing sutures, and the patient was discharged after 2 days of hospitalization with an uneventful postop course. In the patients' further follow ups, no neurovascular complications or signs of infection was reported, while also achieving full range of motion in examination.

## 3. Discussion

Although upper extremity peripheral pseudoaneurysm of the arteries is rare, they can happen iatrogenically and after trauma, especially in upper extremity fractures. Nowadays, the total rate of pseudoaneurysms has increased due to the widespread use of peripheral endovascular procedures from 0.04% before 2000 to 0.7% in 2019 [5, 6]. Arterial catheterization for blood gas, invasive monitoring, and diagnostic and therapeutic endovascular procedures are considered as the most common causes of iatrogenic injury to the brachial artery. Even though it is uncommon, the development of pseudoaneurysms is slow and can cause delayed diagnosis with further possible complications. Pseudoaneurysms, depending on the size, can be asymptomatic or present with symptoms ranging from pressure effect on the adjacent tissues such as pain, swelling, and nerve injury, or ischemic complications due to thrombosis or distal embolization with the management depending on the location, size, and causative pathogenesis of pseudoaneurysm [7, 8].

Table 1 demonstrates reported cases of pseudoaneurysm following supracondylar fracture of humerus after fixation. Most of the reported cases happened in close reduction and fixation with Kirschner wires, and in most cases in the first follow up of the patient, clinical suspicion to pseudoaneurysm formation was not made, and surgeons assumed that swelling and pain contribute to the postsurgery inflammation or postsurgery hematoma which further evaluation and follow ups revealed the diagnosis of pseudoaneurysm.

Asavamongkolkul and Ruangsetakit reported pseudoaneurysm of the brachial artery after supracondylar fracture in a 3-year-old boy. Similar to our case, the patient was treated using Kirschner wire fixation after an open reduction in the operating room. In this case, the patient presented with a slowly growing nonpulsatile soft tissue mass on the medial aspect of the left elbow with audible bruit. However, in contrast to our case, the interval of the initiation of symptoms was much longer, and it was found that the tip of the Kirschner wire was stuck in the pseudoaneurysm that could penetrate the brachial artery in some positions, which was not the case in our study. Also, the patient in their study developed malunion, while ours recovered after surgery without any complications in the follow-up visits [9].

Kemp et al. described an 82-year-old man who was referred to their clinic due to a painless pulsatile soft tissue mass with bruit following a comminuted fracture of the right humerus. He was treated with a hanging cast; however, since the incident, he developed loss of dexterity in his right hand and paresthesia. CT angiography confirmed the presence of a large brachial pseudoaneurysm. Therefore, he was transferred to the operating room for the surgical repair of the pseudoaneurysm. On close inspection during surgery, it was found that the sac contained a sharp fragment of the humerus with a single puncture wound in the brachial artery which was the reason for pseudoaneurysm formation [8].

Moran et al. reported a 65-year-old female who presented with a large pulsatile soft tissue mass in the middle third of the left humerus 6 months after the humeral fracture which was fixed with an intramedullary nail. The patient presented with reduced sensation in the median nerve territory along with mild thenar muscle wasting. A pseudoaneurysm with the largest diameter of 5.8 cm was diagnosed with duplex sonography which was due to a break of proximal locking screw of the intramedullary nail. Although humeral fracture was fixed with an intramedullary nail, the patient developed brachial pseudoaneurysm [7].

It is worth mentioning that although orthopedic references put an emphasis on considering anterior approach for exploration and reconstruction. However, our patient developed iatrogenic pseudoaneurysm of the profunda brachii due to penetration of Kirschner wires used in open reduction and internal fixation through posterior approach. When utilizing posterior approach, persuasion should be taken to avoid neurovascular damage too while using Kirschner wires. Moreover, it should be mentioned that orthopedic surgeons must take the vascular complications in patients' follow-ups as well as other complications such as nerve damage, stiffness, and infection into consideration. This case emphasizes the importance of clinical suspicion to pseudoaneurysm even if the distal pulses were palpated and duplex sonography reveals normal arterial flow. Furthermore, close vascular observation must be done in the setting of well-perfused hand in cases of displaced supracondylar fractures before and after surgery.

## 4. Conclusion

Pseudoaneurysm is a complication that presents itself gradually and can be either asymptomatic or symptomatic, in which any external instrument or fragments of fracture with a sharp end can cause its formation. A high index of suspicion is necessary in cases with humeral fractures for the early diagnosis of pseudoaneurysm where the delayed diagnosis may cause further complications.

## Figures and Tables

**Table 1:** A review of reported cases of arterial pseudoaneurysm after supracondylar fracture of humerus fixation.

Firth author	Age (years)	Interval till initial presentation	Initial physical exam	Diagnosis	Type of fixation	Intraoperative findings agent
Asavamongkolkul and Ruangsetakit [9]	3	7 months	(i) 4 cm mass without erythema or warmth (ii) No pulsation, bruit on auscultation	Brachial angiography	OR via a posterior longitudinal incision and internal fixation with two-crossed K-wire.	Tips of the K-wire were stuck inside the aneurysm.
Chikande et al. [10]	6	11 days	(i) Painful tumefaction of the medial and distal part of the arm (ii) Fever (iii) Weak radial pulse (iv) Normal cutaneous hand perfusion	Duplex ultrasound and CT angiography	CRIF using 3 K-wire, 2 percutaneous from lateral condyle, and one by a mini-approach on medial epicondyle.	Brachial artery perforation without contacts with wires.
Hernandez et al. [11]	7	21 days	(i) Medial mass of the elbow, with no palpable thrill, or audible bruit (ii) Distal radial and ulnar pulses were present	Duplex ultrasound and CT angiography	CR and internal fixation with two lateral K-wires under fluoroscopic control.	Injury may be produced by punction humeral artery during the insertion of the wires.
Cunha et al. [12]	9	12 weeks	(i) Palpable, painless, and pulsatile mass (ii) Distal pulses were symmetric and palpable	Duplex ultrasound	Percutaneously using three K-wire placed divergently in a lateral-to-medial position.	Bone spicula perforating the artery during trauma or the maneuver reduction.
Got et al. [13]	6	5 weeks	(i) Diminished sensation and strength in the radial nerve distribution (ii) Diminished radial pulse (iii) Continued swelling in the antecubital fossa	Soft tissue sonography.	CR and percutaneous pin fixation of the supracondylar fracture with 3 lateral K-wire.	The pseudoaneurysm was a 1 cm segment of the posterolateral wall of the brachial artery.
Tepper et al. [4]	9	22 days	(i) Elbow mass, tender to palpation, and no pulsatile quality (ii) No overt palpable thrill (iii) Normal neurovascular examination	Intraoperative diagnosis was done during hematoma evacuation.	CR and percutaneous pin fixation with 2 K-wire inserted from the lateral condyle and divergent across the fracture site.	Ruptured left brachial artery pseudoaneurysm and a complex longitudinal arterial tear at the fracture site. The fixation pins were not near the pseudoaneurysm and were left in place because the fixation remained stable.
Bhandari et al. [14]	13 years old	2 weeks	(i) An expansile swelling of 5 cm diameter with thrill with normal radial pulse	—	CR and k-wire fixation (4 K-wire)	Brachial artery pseudoaneurysm

CR: closed reduction; CRIF: closed reduction and internal fixation; CT: computed tomography; OR: open reduction; K-wires: Kirschner wires.

## Data Availability

Please contact the corresponding author for any further data.

## Abbreviations

CT: Computerized tomography.
